# Abusive language detection in youtube comments leveraging replies as conversational context

**DOI:** 10.7717/peerj-cs.742

**Published:** 2021-10-08

**Authors:** Noman Ashraf, Arkaitz Zubiaga, Alexander Gelbukh

**Affiliations:** 1 Instituto Politécnico Nacional, CIC, Mexico City, Mexico; 2 Queen Mary University of London, London, United Kingdom

**Keywords:** Context aware abusive language detection, Abusive language detection, YouTube, Natural language processing, Corpus, Deep learning

## Abstract

Nowadays, social media experience an increase in hostility, which leads to many people suffering from online abusive behavior and harassment. We introduce a new publicly available annotated dataset for abusive language detection in short texts. The dataset includes comments from YouTube, along with contextual information: replies, video, video title, and the original description. The comments in the dataset are labeled as abusive or not and are classified by topic: politics, religion, and other. In particular, we discuss our refined annotation guidelines for such classification. We report a number of strong baselines on this dataset for the tasks of abusive language detection and topic classification, using a number of classifiers and text representations. We show that taking into account the conversational context, namely, replies, greatly improves the classification results as compared with using only linguistic features of the comments. We also study how the classification accuracy depends on the topic of the comment.

## Introduction

With the proliferation of social media sites, there is an increase in user-generated content as users can easily post content online and communicate with others. However, some users exploit this possibility to misuse social media platforms by posting abusive content and deliberately affronting others. Duggan (2017) reported that a large number of users on social media have experienced abusive behavior, or have observed cases of harassment directed to other fellows. Research has shown that these events not only lead to mental stress and anxiety in users but, in some cases, individuals end up shutting down their social media accounts and, in extreme cases, even causes individuals to take their own lives (Hinduja & Patchin, 2010; Ashraf et al., 2020; Mustafa et al., 2020). The severity of the consequences of online abuse urges the need to research the development of abusive language detection models (Yin & Zubiaga, 2021).

Over the last few years, there has been an increasing body of research tackling abusive language in fields including Natural Language Processing (NLP), Web Science, and Artificial Intelligence (AI) (Djuric et al., 2015; Hosseinmardi et al., 2015; Ribeiro et al., 2017; Serra et al., 2017). Early work is based on Machine Learning (ML) classifiers such as Support Vector Machines (SVM) and Logistic Regression (LR) with word and char *n*-gram features (Greevy & Smeaton, 2004; Kwok & Wang, 2013; Mehdad & Tetreault, 2016; Yin et al., 2009). They developed regular expressions, contextual features, and predefined abusive words to detect abusive language from sentences. More recent research is based on deep learning models such as Convolutional Neural Network (CNN), Long Short-Term Memory Networks (LSTM), Recurrent Neural Network (RNN), and Bidirectional Long Short-Term Memory (Bi-LSTM) for the detection of abusive language and hates speech (Zampieri et al., 2019a; Wulczyn, Thain & Dixon, 2017; Devlin et al., 2019; Plaza del Arco et al., 2021; Cecillon et al., 2021).

In this paper, first, we discuss certain problems with annotation guidelines for abusive language detection used by other authors and propose improved annotation guidelines for this task, which we consider clearer and more accurate. Then, we present an annotated dataset for abusive language detection in English, consisting of YouTube comments with with contextual information, in particular, conversational context in the form of replies. Our dataset, which we called Context-Aware Abusive Language Detection in YouTube Comments (CAALDYC) dataset, thus allows for leveraging context for the YouTube abusive language detection task. We show that the contextual information is very important for this task, as, we believe, for any short text classification task. To the best of our knowledge, our dataset is the first one of its kind: we are aware of two datasets for English abusive language detection task based on YouTube comments (Obadimu et al., 2019; Mollas et al., 2020), but they do not include the contextual information. All other abusive language datasets we are aware of are not based on YouTube comments or are in other languages; see “Related work”.

In addition to the abusive *vs*. non-abusive labels, the comments in our dataset are annotated with topic labels: politics, religion, and other.

However, these labels are auxiliary, only to allow for some more fine-grained insights in the classification process; we deliberately over-simplified the annotation guidelines for these labels, since a realistic topic classification is outside of the scope of this work; see “Annotation” for details.

Our main contributions can be summarized as follows:

- A new contextual abusive language detection dataset of YouTube comments, along with replies, in which comments are labeled for abusive *vs*. non-abusive language with an auxiliary classification into three topics;
- Refining the annotation guidelines for abusive language detection and topic classification.
- Strong baseline results that provide a benchmark for future research on context-aware abusive language detection in YouTube comments. The baseline results include a number of classical and state-of-the-art classifiers and two text representation techniques;
- A confirmation—on the case study of our YouTube comments corpus—of the intuition of that context information greatly helps in short-text classification;
- Observations on the classification behavior on different topics.

Our dataset, CAALDYC, is freely available for research purposes (https://www.gelbukh.com/resources/caaldyc; last visited: 28-01-2021).

The rest of the paper is organized as follows. “Related Work” gives an overview of abusive-language datasets and classification models. “Problem Statement and Annotation Guidelines” discusses the problem statement and presents an improved definition of the concept of abusive language. “Building the Dataset” describes the development of the dataset. “Benchmarks” presents evaluation of our models. “Results and Analysis” analyzes the experimental results and error analysis. “Discussion” discusses the characteristics and limitations of the dataset. Finally, “Conclusion and Future Work” concludes the paper and outlines future work directions.

## Related work

Research in the area of abusive and hate speech detection has been extended across various overlapping fields. As a result, various public datasets exist for abusive language detection.

### Abusive language datasets

The Smokey-Corpus was the first one published in the abusive language domain that contains 1,222 private messages of the English language and they labeled the corpus into three classes: flame, maybe flame, and okay (Spertus, 1997). Various datasets have been assembled by using Yahoo! portals, particularly related to Finance and News. The Yahoo!-Fin-Corpus of 951,736 comments was developed in English by using the Yahoo! finance portal. The dataset was annotated by using two labels: hate speech and clean (Djuric et al., 2015). Similarly, Yahoo!-Fin-Corpus-2 was created and they applied various deep-learning models on different kinds of syntactic and, embedding features (Nobata et al., 2016). The Twitter-WH-Corpus is one of the most popular corpora and it contains 16,907 tweets. These tweets were labeled into three classes such as racism, sexism, and neither (Waseem & Hovy, 2016). Tweet-NSA-Corpus of 80,000 tweets was created and annotated into four classes: normal, spam, hateful, and abusive (Founta et al., 2018). Davidson et al. (2017) created a dataset from Twitter for hate speech detection and it contains 24,802 tweets. They labeled tweets into three categories: hate speech, offensive language, or neither and used rigorous criteria to annotate their dataset. Wiki-Att-Corpus, Wiki-Agg-Corpus, and Wiki-Tox were released and they were gathered from Wikipedia history (Wulczyn, Thain & Dixon, 2017). Obadimu et al. (2019) analyzed five types of toxicity from YouTube comments. They collected their dataset from pro-and anti-NATO channels on YouTube and assigned toxic scores to each comment using Google’s Perspective API.

A few researchers explored abuse in European languages other than English. To detect cyber-bullying from Dutch posts (Van Hee et al., 2015) created a dataset known as Dutch-Bully-Corpus. It consists of 85,485 cyber-bullying posts from *ask.fm*. Mubarak, Darwish & Magdy (2017) developed a dataset from YouTube in the Arabic language that consists of 1,100 tweets and 32,000 comments. They categorize the dataset into three classes: obscene, offensive, and clean. A Greek-Gazzetta-Corpus consists of approximately 1.6 million comments that are labeled into two classes: accepted or rejected created by Pavlopoulos, Malakasiotis & Androutsopoulos (2017). The data was collected from the news portal Gazzetta. There are several datasets that are associated with shared tasks and often used for multiple languages such as OffensEval for English, Arabic, Danish, Greek, and Turkish (Zampieri et al., 2019b, Zampieri et al., 2020), GermEval 2018 for German (Wiegand, Siegel & Ruppenhofer, 2018), HASOC 2019 for English, German, and Hindi (Mandl et al., 2019), TRAC 2018 to 2020 for English, Bengali, and Hindi (Fortuna et al., 2018), SemEval-2019 task 5 hate Speech detection in Spanish and English (Basile et al., 2019). Table 1 summarizes the details of the existing datasets and their features. For a more exhaustive review of existing datasets for abusive language detection, we refer the reader to Vidgen & Derczynski (2020).

**Table 1:** Summary of datasets on social media to detect abusive language and hate speech.

Platform	Language	Classes	Methods	Size	Results
News site (Sood, Antin & Churchill, 2012)	English	abusive or not abusive	Levenshtein Edit Distance (LED), SVM	6,500	F_1_ 63%
Yahoo! finance (Djuric et al., 2015)	English	hate speech, clean	CBOW, paragraph2vec	951,736	F_1_ 80%
Twitter (Waseem & Hovy, 2016)	English	racism, sexism, neither	character *n*-grams	16,907	F_1_ 73%
Wikipedia (Wulczyn, Thain & Dixon, 2017)	English	attack, non-attack	word-or character-level *n*-grams, LR, MLP	95.1 M	Acc 96%
Twitter (Founta et al., 2018)	English	normal, spam, hateful, abusive	Correlation Coefficients, Cosine Similarity	80,000	F_1_ 73%
Twitter (Davidson et al., 2017)	English	hate speech, offensive language, or neither	*n*-grams, LR	24,802	–
Private messages (Spertus, 1997)	English	flame, maybe flame, okay	DT	1,222	–
YouTube, Reddit (Mollas et al., 2020)	English	binary (ishate *vs*. nohate) and multi-label (violence, directed *vs*. generalized, gender, race, national origin, disability, sexual orientation, religion)	DistilBERT, NNRB	binary: 998, multi: 433	F_1_ 78%, F_1_ 70%
Twitter (Zampieri et al., 2019a)	English	offensive or non-offensive	unigrams, SVM, CNN, BiLSTM	14,100	–
YouTube (Obadimu et al., 2019)	English	five types of toxicity	Latent Dirichlet Allocation (LDA)	–	–
ask.fm (Van Hee et al., 2015)	Dutch	cyberbullying, non-cyberbullying	BOW, SVM	85,485	F_1_ 55%
Gazzetta (Pavlopoulos, Malakasiotis & Androutsopoulos, 2017)	Greek	accepted or rejected	character or word n-grams, CNN, RNN	1.6 M	Acc 97%
Twitter (Mubarak, Darwish & Magdy, 2017)	Arabic	obscene, inappropriate	list-based methods	1,100/32,000	F_1_ 60%
Facebook, Twitter (TRAC2018) (Fortuna et al., 2018)	English, Bengali, Hindi	non-aggressive, covertly aggressive, overtly aggressive	POS, MLP, Ensemble learning	15,000	–
Twitter (GermEval Task2 2019) (Struß et al., 2019)	German	hate, type, implicit/explicit	word embeddings, character n-grams, SVM, LSTM	4,000	F_1_ 76%
Twitter (HateEval at SemEval) (Basile et al., 2019)	Spanish, English	hate, aggression, target	MFC, SVM	19,000	F_1_ 65%

### Approaches to abusive language detection

A number of recent surveys have covered approaches to hate speech and abusive language detection (Fortuna & Nunes, 2018; Schmidt & Wiegand, 2017; Poletto et al., 2020). One of the early works used a supervised classification technique and *n*-gram features to tackle abusive language (Yin et al., 2009). They manually developed regular expressions and contextual features which were used to determine the abusiveness of previous sentences. Most of the basic techniques use predefined abusive words. Sood, Antin & Churchill (2012) recognized that some abusive words might not be abusive in real-life situations. Edit distance metric and abusive word lists were used to improve the detection of profanity which allowed them to get non-normalized terms such as “*@ss*” or “*sh1t*”. Additionally, this was the first time crowdsourcing was used to annotate abusive language. Amazon Mechanical Turk workers labeled 6,500 comments from the internet into two classes as abusive or not abusive. Waseem & Hovy (2016) used extra linguistic-based features with the combination of character *n*-grams to identify hateful text. Wulczyn, Thain & Dixon (2017) explored ML classifiers with word and character *n*-gram approaches and achieved the highest accuracy of 96.59%. A logistic regression model was prepared with *L2* regularization to differentiate between these categories and they discussed major issues for accurate classification.

A combination of lexical resources and parser features was also used to detect the offensive language in YouTube comments to protect teenagers (Chen et al., 2012). However, they did not provide a strict definition of offensive language. They used a support vector machine classifier with *n*-gram features, regular expressions and dependency parse features. Spertus (1997) used decision-tree to analyze feature-based rules and predict 64% of the samples as flames and 98% as non-flames. The best model achieved 90.55% accuracy and surpassed the state-of-the-art accuracy by ten points. Van Hee et al. (2015) explored three text representation approaches: word *n*-grams, character *n*-grams, and sentiment-lexicon features and obtained F1 score of 55.39%. Obadimu et al. (2019) used Latent Dirichlet Allocation (LDA) topic modeling technique to identify positive and negative topics such as “*Alliance*” and “*Profanity*”. They achieved a precision score of 98.24% and a recall of 94.34%. They explored whether the offence was targeted or not, and the target was individual, a group, or otherwise.

Recently, deep learning and graph-based approaches were explored to detect abusive language and hate speech (Zampieri et al., 2019a; Wulczyn, Thain & Dixon, 2017; Djuric et al., 2015; Cecillon et al., 2021). Zampieri et al. (2019a) implemented Bi-LSTM, and CNN to perform the experiments and best micro-F_1_ scores of 80% and 69% were obtained on the first two levels using CNN. However, the same score of 47% was achieved by two classifiers: CNN and Bi-LSTM in the last level. GRU recurrent neural network (RNN), RNN with attention mechanism, convolutional neural network (CNN), and detox were implemented on Greek-Gazzetta-Corpus (Wulczyn, Thain & Dixon, 2017). Djuric et al. (2015) used the paragraph2vec method to classify user comments as abusive or clean. They applied various techniques but bag-of-words outperformed other methods and achieved an accuracy of 78.89%. Cecillon et al. (2021) used graph embedding approaches that can learn representations of graphs from online messages. This study discussed aspects of graph structure more accurately. They compared two types of categories such as node *vs*. whole-graph embeddings and achieved 89.16% F_1_-measure through Graph2vec.

Finally, researchers are using pre-trained transformer models such as Bidirectional Encoder Representations from Transformers (BERT), RoBERTa, ALBERT and GPT-2 to detect hate speech (Lan et al., 2019; Radford et al., 2019; Devlin et al., 2019; Srivastava et al., 2021). Vashistha & Zubiaga (2021) applied BERT, XLM and BETO models and achieved promising results. Zhang et al. (2020), Muennighoff (2020) used various transformer models: Visual BERT, ViLBERT, VLP, UNITER, LXMERT, VILLA, ERNIE-Vil, and Oscar for hateful meme detection.

## Problem statement and annotation guidelines

Abusive language has many terms and various standards on the web that can influence what is treated as abusive language (Chandrasekharan et al., 2018). In the context of natural language processing, the term *abuse* encloses various types of negative expressions. Mishra, Yannakoudakis & Shutova (2019) define the term as *“any expression that is meant to denigrate or offend a particular person or group”*. Profanity, hate speech, and derogatory language is referred to as abusive by Nobata et al. (2016), while Mishra et al. (2018) used the abusive term in the context of sexism and racism. Waseem et al. (2017) categorized the term abuse generally dependent on *explicitness* and *directness*. *Explicit* abuse comes as harsh words or dangers, whereas *implicit* abuse has an indirect appearance described by uncertain terms such as sarcasm. *Directed* abuse focuses on a specific individual instead of generalized abuse.

Therefore, one of the major problems with the existing definitions is that they are ambiguous and overlapping. In a simple text, without context, for example, “you get angry too easily” is considered abusive according to some of the above-given definitions, however, it is criticism and its intention is not to abuse but help people to improve their behavior or deficiencies. Another obstacle hindering detection of abusive content is the brevity of social media content, often misinterpreted by annotators if they do not have access to the context (Chatzakou et al., 2017). A specific piece of text such as sarcasm can be wrongly classified as abusive or harmful when seen in isolation, but taking into account earlier discussion one can see that in fact, it is not profanity (Founta et al., 2018). For instance, in the Friends TV series, one of the actors said “what the fuck you doing” to another actor. This sentence contains a profanity word; however, the previous sentence in the same turn “I still have feelings for you” shows that in fact, this sentence was friendly. Such phenomena make the abusive language detection task difficult and creating a standard dataset is time-consuming and labour expensive. Because of this, to date, existing solutions are far from being sufficient to deal with the problem (Musaddique, 2017; Robertson, 2017).

Thus, for our dataset, we instructed the annotators as follows:

*Abuse is any form of expression that (1) addresses another person, group or community, (2) is derogatory, sexist, vulgar or profane, and (3) refers to human flaws, intends to offend a person or a group, or implies condescension or victim-blaming*.

For instance, consider these two examples from our corpus:^1^ 1We keep the orthography of the comment from our corpus. “What a dumb bitch, keep her in jail please for just being stupid” (this comment contains a derogatory word and it is also about human identity); “White people suck” (it contains a profanity word and also offends a group of people: White). Both of these examples are abusive as per our definition of abusive language. However, “you get angry too easily” is not abusive according to our definition of abuse, while, as we have discussed above, some of the existing definitions identify this example as abusive.

Explicit abuse (direct abuse) is relatively simple and can be detected easily with machine-learning techniques, however, this is not the case with implicit abuse (generalized abuse) (Dadvar et al., 2013; Waseem & Hovy, 2016). To address the issue of implicit abuse it is extremely important to have a context. For this purpose, our annotators had access to all information included in the dataset, such as comments, replies, video, video title, and the original description. The annotators could consider this additional information or could ignore it (such as lengthy videos) as they preferred; see “Building the Dataset” for details.

## Building the dataset

This study presents a novel dataset of YouTube comments for abusive language detection. This dataset will allow working with additional information of comments containing abusive or non-abusive text. Most existing datasets solely rely on keyword-based search to retrieve relevant content, hence restricting abusive content that contains those predefined keywords. In addition, they did not provide extra information related to the main abusive text. Therefore, these datasets provide limited information that doesn’t enable researching context-aware abusive language detection. To further research in this direction, we introduce a novel method for collecting a dataset to solve this limitation. In this section, we discuss data collection and processing, describe the annotation process of YouTube comments, characteristics of the dataset, and dataset standardization.

### Data collection and processing

First, we manually selected 29 YouTube videos based on topics: politics, religion, and other. These are videos published by popular sources like BBC, CNN, or TV shows like Saturday Night Live and, due to their popularity, have a large number of comments and replies. The detail of these videos is available for analysis (https://github.com/Noman712/contextual-abusive-language-detection/blob/main/dataset/Data_Collection_Videos.xlsx; last visited: 22-01-2021). We collected all the data that are related to these videos such as ID, title, comments, replies of comments, likes, date, and time. Initially, our data collection led to more than 160,000 comments associated with these videos, retrieved through the YouTube data API (https://developers.google.com/youtube/v3; last visited: 28-01-2021). YouTube API has a restriction on per day pings, and we were able to ping 10,000 times per day. Most of the comments were published between 2016 and 2017 and extracted for this study from December 2019 to January 2020. Moreover, we have included all the replies to the comments. The extracted comments and replies of each video were stored in separate CSV files in chronological order. Next, we converted the dataset into a single CSV file containing the columns: video-URL, title, comment, replies, date, and time. Data was grouped and sorted together based on the date and time of the video. Finally, comments that do not have replies were removed for two reasons: (i) they were not providing extra information about comments, (ii) to avoid difficulties in the annotation process because they were not fulfilling our annotation requirements. After removing these comments, we were able to extract 18,794 comments that have replies. However, this is not the final statistics of the comments and replies because these comments are in various languages such as Spanish and Hindi. As a result, we only selected comments which were in English language; see “Dataset Statistics” for details.

### Annotation

We prepared a set of annotation guidelines (see “Problem Statement and Annotation Guidelines”) to assist the annotation process of the proposed CAALDYC dataset, which was iteratively revised following internal discussions between the annotators. The annotations were performed by three annotators (A1, A2, and A3). They all have a good command of the English language and are experienced in social media and NLP research. These annotators are from a computer science background and have a minimum qualification of Master’s degree; one of the annotators is an author of this paper, and two others are from Chang Gung University, Taoyuan, Taiwan. Two annotators are male, and one is female. They are from the same continent: Asia, and have the same faith: Islam. In the beginning, two annotators did a pilot annotation test for the first 100 comments and replies. These annotations were discussed, and a guideline for re-annotation was developed from the beginning for better quality and consistency. Using these guidelines, all annotators annotated the complete dataset, which was 2,304 comments. Finally, annotator A3 provided annotations for cases where A1 and A2 had disagreed, enabling to break the tie and determine the final annotations.

Since this research focuses on context-aware abusive-language detection, annotators were asked to assign one of the two labels (abusive or non-abusive) by taking into account the additional information of the comments. In addition to being annotated with abusive language detection labels, comments in our dataset are classified into topics: politics, religion, and other. Some of the comments intersect in topics; however, for the sake of simplicity, we ask the annotators to choose the most relevant topic, which is, of course, an oversimplification. These auxiliary topic classification is essential to understand what type of language is used and the behavior of the classification algorithms on different topics. It also enables us to conduct topic classification experiments, determining the difficulty of each class.

A hierarchical annotation scheme was used to divide our dataset into two tasks: abusive *vs*. non-abusive (task 1) and its topic classification of abusive language (task 2).

#### Task 1: abusive language detection

- **Abusive:** *Abuse is any form of expression that (1) addresses another person, group or community, (2) is derogatory, sexist, vulgar or profane, and (3) refers to human flaws, intends to offend a person or a group, or implies condescension or victim-blaming*.
- **Non-Abusive:** Any comment that is not in the abusive category.

#### Task 2: topic classification of abusive language

- **Politics:** Comments in which abusive language is used under the guise of political affiliation.
- **Religion:** Comments in which abusive language is used under the guise of religion, including harassment or humiliating messages (https://en.wikipedia.org/wiki/Religious_abuse; last visited: 07-05-2021).
- **Other:** Any comment that is not in the religion and politics topic.

Table 2 illustrates the examples of comments and their replies. From the examples, we can see that labeling was done based on the comments and contextual information rather than abusive words. For instance, the fifth example from Table 2 shows that comment is not abusive, but replies are. Hence, the example is marked non-abusive because the labeling is based on additional information rather than solely on abusive words. The resulting CAALDYC dataset is freely available for research purposes (https://www.gelbukh.com/resources/caaldyc; last visited: 28-01-2021).

**Table 2:** Examples from dataset containing comment and replies from abusive *vs*. non-abusive classes.

Comment	Replies	Classes
racist son of a bitch	“A very punchable face indeed”, “He was wearing a heavy jacket that was protocol not racism”, “Lol chill dude he is an actor”	Abusive
What a disrespectful idiot. Hope he’s serving decades; one less fuck idiot off the street.	“Ok liberal Pussy”	Abusive
haha shut her basic white as down	“How is he a racist, you fuck muppet?”	Abusive
apparently Donald can’t take this joke	“Jacob Medors a ton of people like to rape others, deal with it.”, “swaggytoast Well, a ton a people enjoy it, so deal with it”	Non-abusive
Sometimes the best hammer in the court is the mouth	“He’s sucking the judge’s dick and doing it wrong which is why the judge is piss”	Non-abusive

### Inter-annotator agreement

We computed Inter-Annotator Agreement (IAA) by using Cohen’s Kappa Coefficient (Cohen, 1960), which led to a kappa coefficient of 74%, indicating substantial agreement and with an IAA which is in line with previous work (Founta et al., 2018).

### Dataset statistics

Tables 3–5 depict the characteristics of the comments and their replies in our dataset after normalization in abusive and non-abusive class based on their respective topics: politics, religion, and other. It also shows the distribution of words and average words per comment and replies in their respective classes and statistics about replies such as average replies per comment. Comments in our dataset have a total vocabulary size of 48,757 words, and replies have a vocabulary size of 240,870 words. The dataset has an average of 3.46 replies in the abusive class and 1.88 in the non-abusive class. For each comment, we have at least one reply. The average word length of each comment is approximately 23 words and around 20 words in their respective class. In comparison, replies have approximately 171 words in abusive class and around 40 words in non-abusive class.

**Table 3:** Statistics of dataset.

Classes	Comments	Replies	Words	Words/Com	Rep/Com	Rep words	Rep Words/Rep
Abusive	1,133	3,924	25,858	22.80	3.46	193,441	170.58
Non-abusive	1,171	2,215	22,899	19.52	1.88	47,429	40.43

**Table 4:** Statistics of topics.

Classes	Topics			Total
	Politics	Religion	Other	
Abusive	225	219	689	1,133
Non-abusive	161	308	702	1,171

**Table 5:** Statistics of each topic class.

Topic	Class	Comments	Words	Avg. words	Total comments
Politics	Abusive	225	4,845	21.43	386
	Non-abusive	161	2,865	17.79	
Religion	Abusive	219	6,050	27.62	527
	Non-abusive	308	7,553	24.44	
Other	Abusive	689	14,963	21.71	1391
	Non-abusive	702	12,481	17.75	

## Benchmarks

To further analyze our CAALDYC dataset and the reliability of our annotations, we performed a set of baseline experiments on several machine and deep learning classifiers. Our dataset includes information such as video, video description, video title, and replies of the comments; however, for the sake of experiments, we used comments and replies. we evaluated our models using Recall (R), Precision (P), and macro F1-measure. These machine and deep learning classifiers have shown competitive performance for several NLP tasks (Devlin et al., 2019; Kim, 2014; Hochreiter & Schmidhuber, 1997; Breiman, 2001; Kohavi, 1995; Bashir et al., 2019; Khan et al., 2021; Butt et al., 2021b; Karande et al., 2021; Ashraf et al., 2021; Ameer et al., 2021; Butt et al., 2021a).

### Preprocessing

We used various pre-processing methods to normalize the CAALDYC dataset. First, NLTK (https://www.nltk.org; last visited: 28-01-2021) library was used to remove stop words and to convert letters to lower case. Similarly, tweet-preprocessor (https://pypi.org/project/tweet-preprocessor/; last visited: 28-01-2021) library was used for removing punctuation marks (such as exclamation marks or single and double quotation marks), digits, URLs, and emoji. Moreover, we replaced contracted words such as *“I’m”* to *“I am”*, *“isn’t”* to *“is not”*. Finally, to ensure that all the comments and replies have equal length in our dataset, we padded short comments with dummy words. Pad sequences function from Keras (https://keras.io/; last visited: 28-01-2021) library was used to perform this functionality. The maximum length of each sequence was set to 24 during GloVe features extraction as the average length of comments and replies were 23 words per sentence and *n*-gram features were extracted from the whole vocabulary.

### Features extraction

GloVe (Pennington, Socher & Manning, 2014) pre-trained model was used to convert words into 300 dimensions vectors from the CAALDYC dataset. YouTube comments and replies were informal and there was a high probability that some words are missing in the GloVe dictionary. So, we decided to add a random uniform distribution of 300 dimensions between [−0.1, 0.1] to comments and replies if they did not have a vector from the GloVe dictionary. Word *n*-grams for our abusive-language detection task are based on *n*-grams taken from the YouTube comments. The *n*-gram refers to a sequence of words (or tokens) from sentences, paragraphs, and documents. To extract the most relevant terms from documents (Ramos, 2003), we used the TF-IDF (term frequency—inverse document frequency) weighting scheme.^2^ 2We used scikit-learn TF-IDF vectorizer for implementation of the *n*-gram model considering the following parameters: use idf = True, smooth idf = True, number of features (Max) and the other than these with default values. https://scikitlearn.org/stable/; last visited: 28-01-2021

Initially, we completed the experiments with YouTube comment vectors without using replies vectors. Likewise, for *n*-gram features, we completed the experiments without the vocabulary of replies. In the second phase, we averaged the embeddings of comments with replies vectors and used these vectors as an input for the classifier. During the extraction of *n*-gram features, we just concatenated replies text with their respective comments and converted it into a single string and used the overall vocabulary of comments and replies to extract *n*-gram features.

### Machine learning classifiers

We used eight machine-learning algorithms: Logistic Regression (LR), Multilayer Perceptron (MLP), Adaboost, Random Forest (RF), Support Vector Machine (SVM), Naive Bayes (NB), Decision Tree (DT), and VotingClassifier for abusive language detection (task 1) and topic classification (task 2). We used the scikit-learn library (Cournapeau, 2007) for the implementation of all machine learning models, and for the experiments, default parameters were set across all ML models. In addition, word *n*-gram features with the help of TF-IDF weighting scheme were used as input to train our ML models (see “Features extraction”). For topic classification, one-*versus*-rest framework was used, which trained a separate classifier for each class. The class label with the highest predicted probability across all classifiers is assigned to each comment in this framework.

### Deep learning classifiers

In this study, we used two neural networks models such as 1-Dimensional Convolutional Neural Network (1D-CNN) and Long Short-Term Memory Networks (LSTM) with additional max-pooling and attention layers for abusive language detection (task 1) and topic classification (task 2). In NLP and opinion mining tasks (Sidorov et al., 2012), these classifiers achieved state-of-the-art performance, and these models have been used for multiple studies in abusive and hate speech detection (Zampieri et al., 2019a; Wulczyn, Thain & Dixon, 2017; Devlin et al., 2019; Plaza del Arco et al., 2021).

Vectors built from the GloVe model were used as input to train our deep learning classifiers. For both tasks, additional layers such as max-pooling and attention were added in our deep learning models to improve the results. In max-pooling layer we consider pool size to “1”, strides to “1”, padding to “valid”, and data format to “channel last”. For attention layer, SeqSelfAttention layer with “sigmoid” activation was used from Keras self-attention library (https://pypi.org/project/keras-self-attention/; last visited: 07-05-2021). Tenfold cross-validation was used for the calculation of results by taking mean accuracy of ten iterations. Additionally, dropout layers and early stopping by loss parameter were added to avoid overfitting. The remaining deep learning parameters for both tasks are presented in Tables 6 and 7.

**Table 6:** Deep learning parameters for abusive language detection.

Parameter	1D-CNN	LSTM
Epochs	100	35
Optimizer	Adam	Adam
Loss	mean squared error	mean squared error
Learning rate	0.0001	0.0001
Regularization	0.001	–
Bias regularization	0.0001	–
Validation split	0.1	0.1
Dropout	0.2	0.2
Early stopping	validation loss (0.1)	validation loss (0.1)
Dense layers activation	tanh	tanh
Last layer activation	Sigmoid	Sigmoid

**Table 7:** Deep learning parameters for topic classification.

Parameter	1D-CNN	LSTM
Epochs	100	35
Optimizer	RMSprop	RMSprop
Loss	categorical crossentropy	categorical crossentropy
Learning rate	0.0001	0.0001
Regularization	0.001	–
Bias regularization	0.0001	–
Validation split	0.1	0.1
Dropout	0.2	0.2
Early stopping	validation loss (0.25)	validation loss (0.1)
Dense layers activation	elu	elu
Last layer activation	softmax	softmax

## Results and analysis

Tables 8 and 9 present our results for classifying comments into abusive and non-abusive class (task 1) while Tables 10 and 11 show our results for the topic of the text (task 2).

**Table 8:** Abusive-language detection using comments dataset.

Features (set)	Data	Classifiers	Accuracy	Precision	Recall	F_1_
*n*-gram	comment	LR	83.91	91.35	74.34	81.94
		MLP	78.71	80.04	75.66	77.75
		Adaboost	88.46	95.13	80.68	87.29
		RF	87.86	93.93	80.51	86.64
		SVM	83.57	91.90	73.01	81.35
		NB	68.57	66.40	73.18	69.54
		**DT**	**88.38**	**90.28**	**85.71**	**87.87**
		VotingClassifier	86.30	92.27	78.74	84.93
GloVe	comment	1D-CNN	89.03	90.84	86.58	88.55
		**1D-CNN + MP**	**90.28**	**87.73**	**92.15**	**89.86**
		1D-CNN + ATT	89.46	89.78	88.80	89.24
		LSTM	88.16	88.98	86.86	87.84
		LSTM + MP	88.51	91.23	85.00	87.93
		LSTM + ATT	90.89	87.56	85.62	89.11

**Note:**

The best result in each row is in bold.

**Table 9:** Abusive-language detection using comments + replies dataset.

Features (set)	Data	Classifiers	Accuracy	Precision	Recall	F_1_
*n*-gram	comment + replies	LR	85.78	89.24	80.86	84.81
		MLP	83.18	82.63	83.42	83.00
		**Adaboost**	**92.32**	**94.60**	**89.59**	**91.96**
		RF	89.64	91.74	86.77	89.15
		SVM	86.30	90.15	81.04	85.32
		NB	64.71	59.59	88.00	71.05
		DT	88.25	88.30	87.83	88.02
		VotingClassifier	88.94	89.18	88.27	88.70
GloVe	comment + replies	1D-CNN	89.07	89.21	88.61	88.85
		1D-CNN + MP	90.68	90.86	90.21	90.48
		**1D-CNN + ATT**	**91.76**	**90.96**	**92.50**	**91.68**
		LSTM	87.51	87.82	86.68	87.21
		LSTM + MP	87.51	88.42	85.88	87.10
		LSTM + ATT	88.46	89.08	87.38	88.15

**Note:**

The best result in each row is in bold.

**Table 10:** Topic classification using comments dataset.

Features (set)	Data	Classifiers	Accuracy	Precision	Recall	F_1_
*n*-gram	comment	LR	77.76	88.70	62.46	67.88
		**MLP**	**84.31**	**83.30**	**78.29**	**80.22**
		Adaboost	82.31	84.77	73.26	77.21
		RF	83.74	87.72	74.40	78.92
		SVM	83.74	88.27	74.12	78.75
		NB	74.60	75.87	61.74	64.87
		DT	80.23	81.61	70.66	74.34
		VotingClassifier	82.92	86.67	72.56	77.02
GloVe	comment	1D-CNN	70.13	67.56	49.26	49.06
		1D-CNN + MP	70.82	71.11	49.98	49.36
		1D-CNN + ATT	70.43	81.60	49.90	50.06
		LSTM	68.57	67.40	46.47	45.53
		LSTM + MP	68.05	62.44	45.72	44.23
		**LSTM + ATT**	**76.11**	**75.08**	**62.64**	**65.08**

**Note:**

The best result in each row is in bold.

**Table 11:** Topic classification using comments + replies dataset.

Features (set)	Data	Classifiers	Accuracy	Precision	Recall	F_1_
*n*-gram	comment + replies	LR	83.92	91.89	71.98	77.36
		**MLP**	**91.50**	**93.26**	**86.36**	**89.13**
		Adaboost	90.16	91.51	85.37	87.81
		RF	89.60	92.87	82.87	86.42
		SVM	89.81	93.44	82.67	86.67
		NB	77.02	76.81	62.94	64.75
		DT	86.56	87.21	79.89	82.57
		VotingClassifier	88.21	91.70	79.13	83.07
GloVe	comment + replies	1D-CNN	85.60	84.53	78.58	80.72
		1D-CNN + MP	85.73	84.61	78.74	80.81
		**1D-CNN + ATT**	**86.43**	**84.66**	**80.94**	**82.43**
		LSTM	81.31	78.64	69.98	71.47
		LSTM + MP	81.27	79.89	70.18	71.53
		LSTM + ATT	81.92	79.84	72.36	74.43

**Note:**

The best result in each row is in bold.

**Figure 1:**
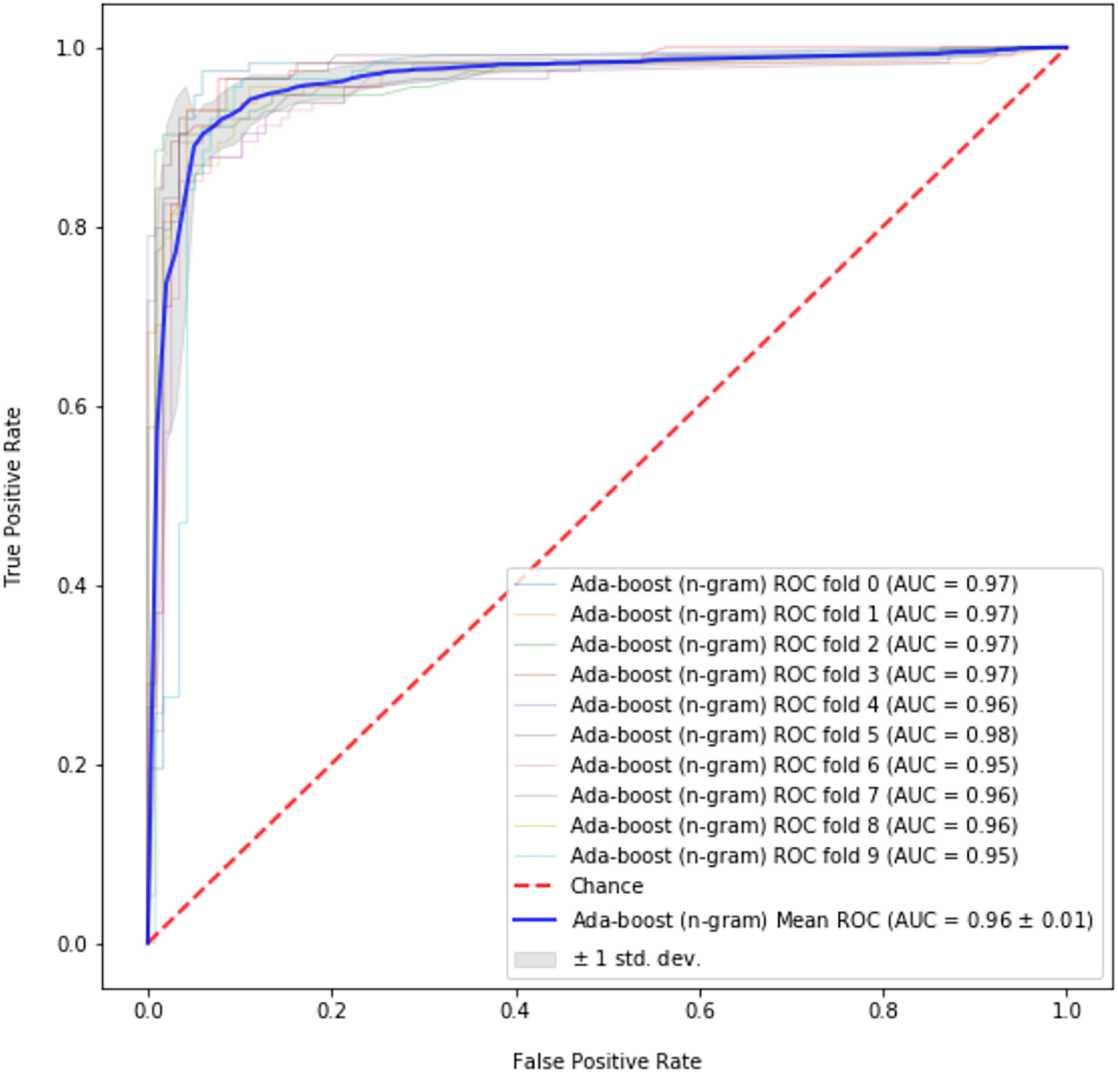
ROC curve for best performing model (Adaboost) on abusive-language detection.

**Figure 2:**
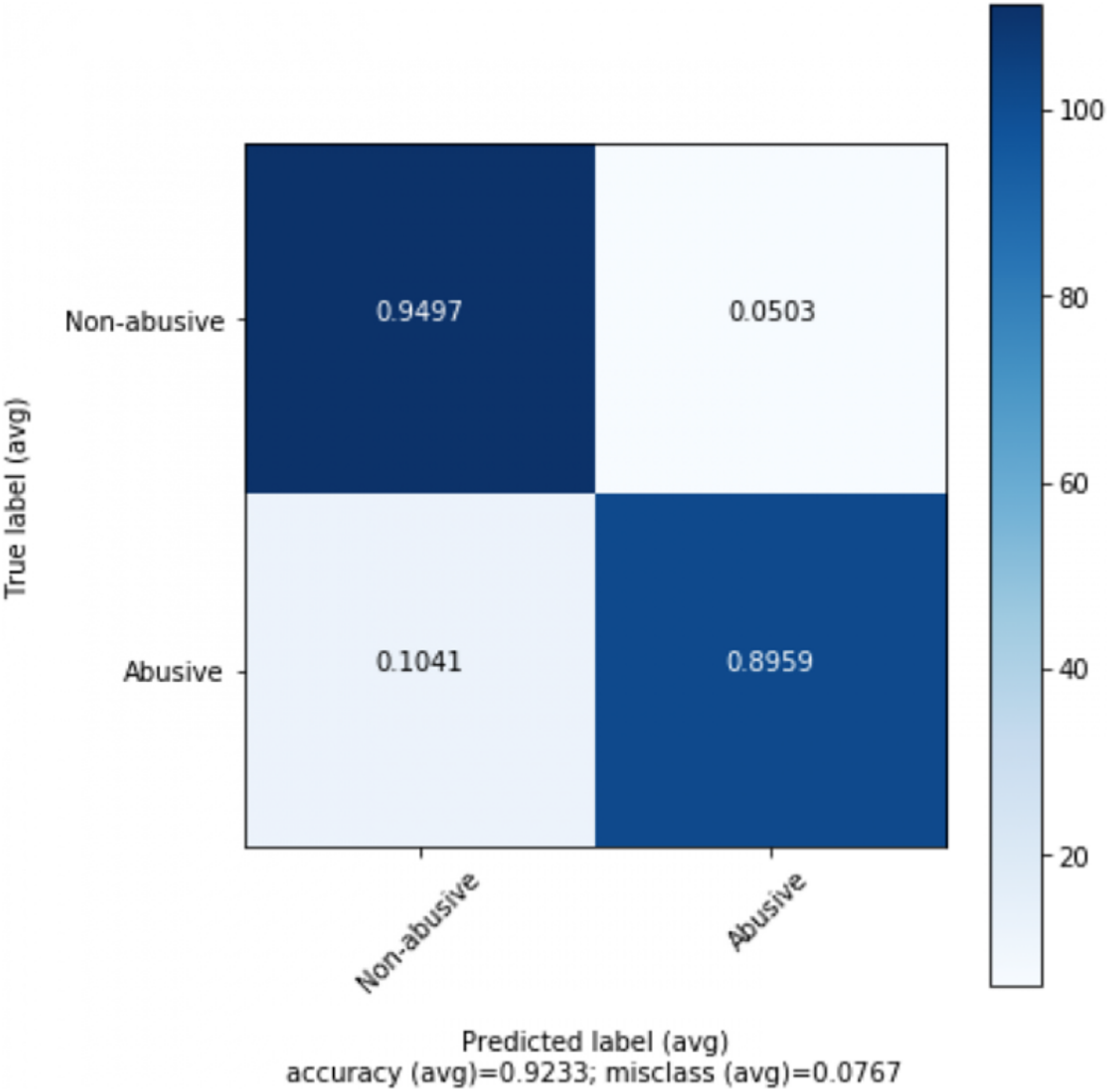
Confusion matrix for the best performing model (Adaboost) for abusive-language detection.

**Figure 3:**
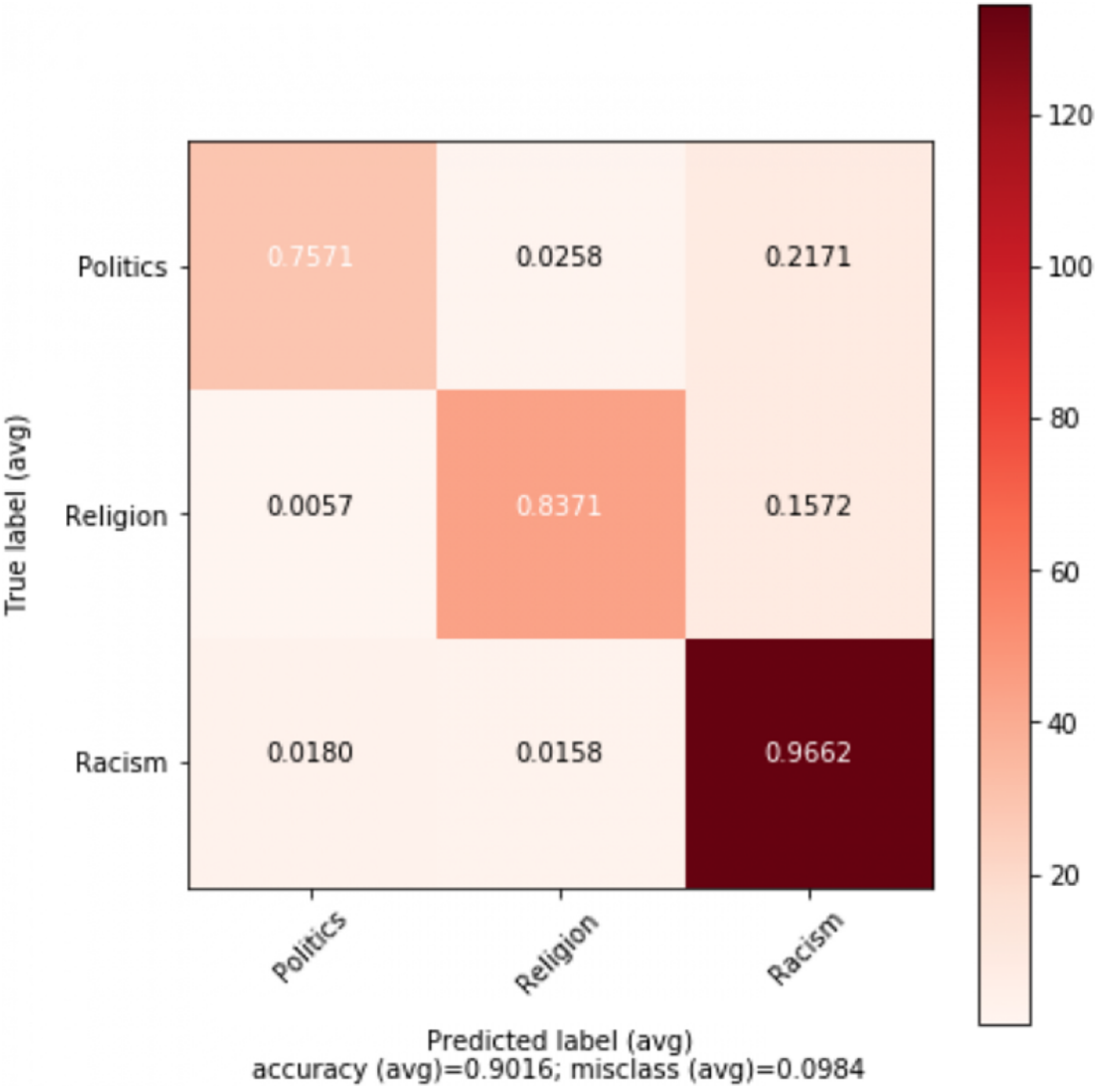
Confusion matrix for the best performing model (MLP) for topic classification.

The best results are obtained for abusive language detection (task 1) using *n*-gram features and GloVe vectors with the help of contextual information. Adaboost classifier achieves an accuracy of 92.32% and F1 score of 91.96% using *n*-gram features while 1D-CNN with attention layer achieves an accuracy of 91.76% and F1 score of 91.68% using GloVe embeddings. Likewise, we achieve the best results for topic classification (task 2) with MLP classifier using *n*-gram features and contextual information. It achieves an accuracy of 91.50% and F1 score of 89.13%. In the tables, the best scores are in bold. Figure 1 shows the tenfold cross-validation mean accuracy across all models, and ROC curves of Adaboost and SVM classifiers on abusive-language detection while Figs. 2 and 3 depict confusion matrix for abusive language detection and topic classification. Interestingly, we observe that variants of our methods using context (*i.e.*, replies) consistently outperform their counterparts ignoring context, hence demonstrating the importance of leveraging context for abusive language detection.

**Figure 4:**
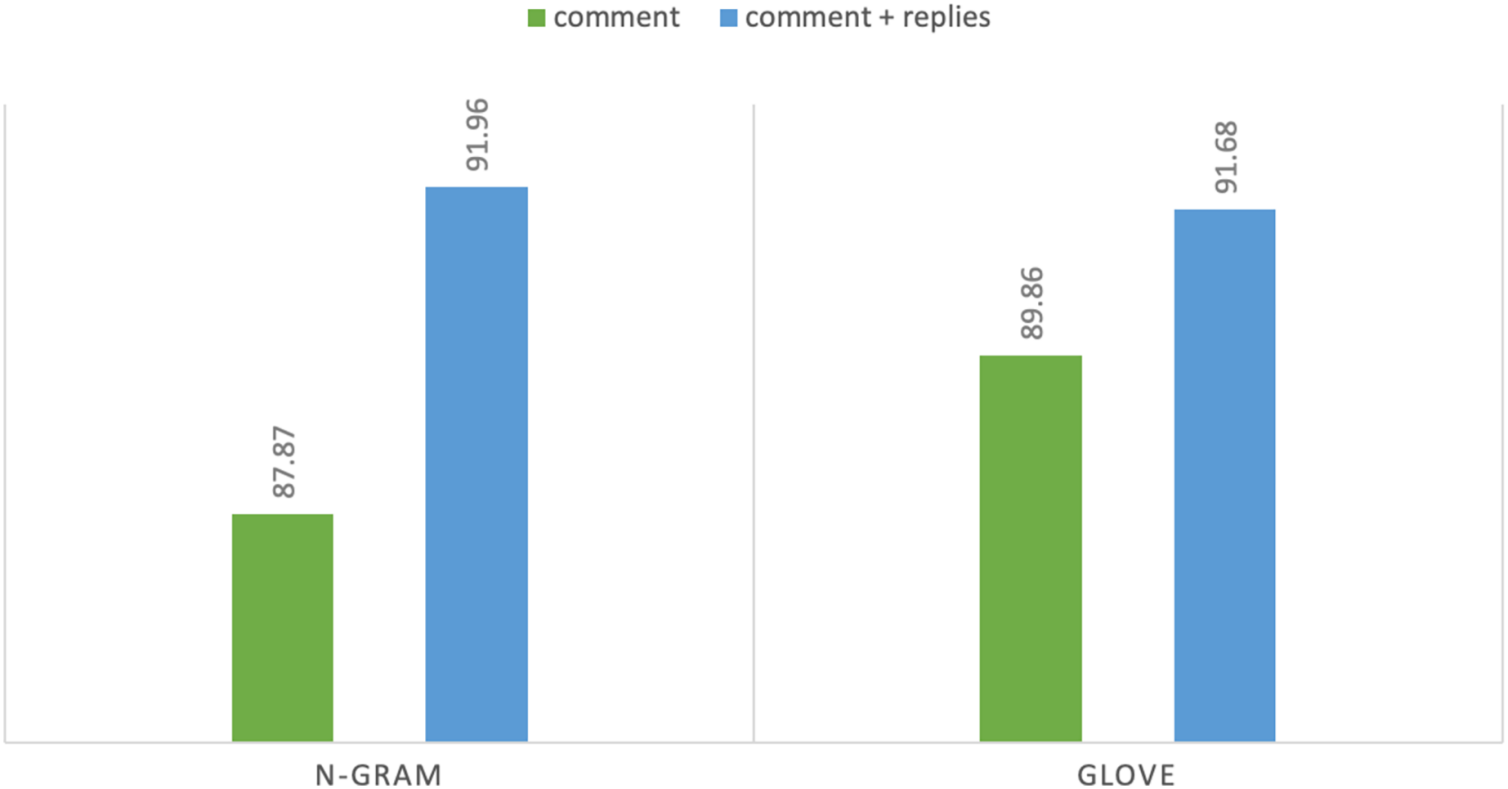
F_1_-measure with two text representations for abusive-language detection.

**Figure 5:**
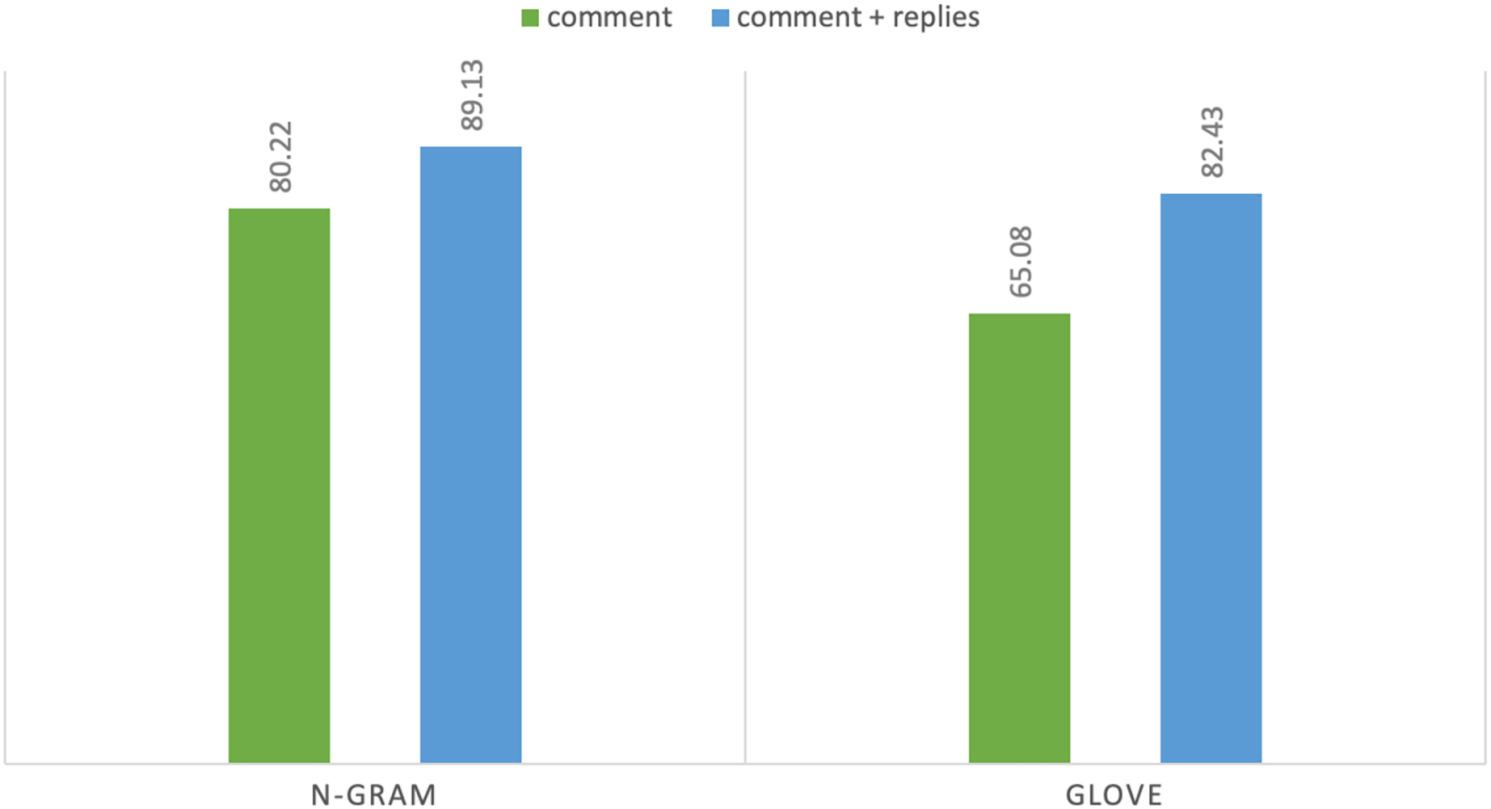
F_1_-measure with two text representations for topic classification.

*N*-gram features showed consistent result improvements on both tasks and all baseline models with extra-linguistic information of YouTube comments. In task 1 using *n*-gram features, DT achieves highest F1 score of 87.87% on comments while Adaboost achieves F1 score of 91.96% using comments and their context: replies. Hence, the difference between the highest F1 score is 4.07% that shows the importance of extra-linguistic information. Moreover, discriminative models (DT, SVM, etc.) perform better than generative classification models such as NB and achieve satisfactory results. Notably, in our experiments from machine learning classifiers, DT and Adaboost achieve the highest accuracy and F1 score. Both of these classifiers are supervised machine learning algorithms and are mostly used for classification purposes. Similarly, in task 2 MLP achieves highest F1 score of 80.22% on comments and achieves F1 score of 89.13% with contextual information. We can see a sharp improvement in results when we utilize additional linguistic information. In the case of task 2 the difference between the highest F1 score is 8.91%. MLP is a deep learning algorithm, and it is widely used for solving problems that require supervised learning. Figures 4 and 5 show the comparison of highest achieved scores using *n*-gram features and GloVe vectors for abusive-language detection and topic classification.

Overall, GloVe vectors on deep-learning models performed better than *n*-gram features without extra-linguistic information and have higher F1 scores than machine learning classifiers. In our experiments, 1D-CNN performs better than LSTM. It is because convolutional neural networks perform well with pre-trained vectors. In addition, CNN has been widely used because it can automatically extract relevant and distinctive features efficiently as well as CNN is highly accurate and computationally efficient as compared with feed-forward neural networks (Kim, 2014, O’Shea & Nash, 2015). 1D-CNN with max-pooling layer achieved F1 score of 89.86% on comments using GloVe vectors, and it is 2.57% higher than *n*-gram features. Although, GloVe pre-trained word embeddings achieve the highest results for abusive language detection (task 1) without extra-linguistic information, however, it was not able to achieve the highest results with contextual information. With additional information, GloVe vectors achieved F1 score of 91.68% which is slightly less than the *n*-gram features. The leading cause of not improving the results is aggregating the vectors of replies with comment vectors because in longer sentences, the meaning of the vector will be dominated by common words such as “like”,“do” and “how”. Another issue would be the dilution of the vector representations because the pre-trained word embeddings are not contextualized. Therefore, even different sentences with the exact words have identical embeddings. For instance, “dog bit John” and “John bit dog” embeddings will be identical. Finally, it is possible that abusive words are not present in text representations that are essential to detect abusive language. Some abusive words have special symbols such as “F**k” that could be missed as out-of-vocabulary. Therefore, further research is needed by applying contextual embeddings and transformers to see if that can enhance the performance of the models. Hence, our results align with state-of-the-art work in the machine and deep learning for abusive language detection.

In addition, several experiments were performed on each of the topics to detect abusive language. The result shows that abusive language is challenging to detect when linked to religion, while politics and other are easier topics. Other topic achieves F1 score of 91.74% with Adaboost. In contrast, topics religion and politics achieve F1 score of 85.48% and 89.6% respectively with SVM. Table 12 shows best results achieved on topics: politics, religion, and other.

**Table 12:** Results by topic, with two text representations. Precision P, Recall R, and F_1_ measure are shown for the abusive class. Bold (italic) stands for the best (worst) result in each row, *i.e*., among the topics.

Features (set)	Data		Politics	Religion	Other
			SVM	SVM	SVM
GloVe	comment + replies	Acc	*87.33*	*87.47*	**89.29**
		P	85.68	*82.94*	**88.32**
		R	**94.28**	*88.57*	*90.42*
		F_1_	**89.67**	*85.48*	*89.28*
*n*-gram features	comment + replies		**RF**	**RF**	**Adaboost**
		Acc	87.32	*86.75*	**92.09**
		P	*87.34*	*89.86*	**94.55**
		R	**92.09**	*77.22*	*89.26*
		F_1_	89.45	*82.62*	**91.74**

Finally, features based on an abusive lexicon (Wiegand et al., 2018) were used to assign weights to all the words in our dataset. Only positive weights were used from the lexicon because they appear to be more abusive. Table 13 shows abusive words and their respective weights based on text topic: politics, religion, and other. Overall, religious comments are more aggressive and abusive, followed by other class. Politics fell behind other class and yielded the least abusive words.

**Table 13:** Highest-weighted abusive words in each topic.

Religion		Politics		Other	
Word	Weight	Word	Weight	Word	Weight
disgusting	3.49	hypocrite	2.41	scum	3.19
moron	3.46	sexist	2.10	horrible	3.18
bastard	3.39	spineless	1.49	crap	2.65
bitch	3.26	incompetent	1.41	brat	2.53
stupid	3.19	cocky	1.33	slut	2.49
ass	3.07	sheeple	1.33	twat	2.28
idiot	3.03	obnoxious	1.31	cunt	2.27
filth	2.77	puppet	1.18	slimy	2.19
scum	2.52	pitiful	1.06	prank	2.07
stinky	2.51	hairy	0.95	faggot	2.05

### Error analysis

To understand the performance of our classifiers, we manually examine a set of erroneously classified comments. We identified two types of errors: type I and type II. In type I errors, comments are manually labeled as abusive but classified as non-abusive by our classifiers. For instance, “Girl Fuk You Judge Boi Im Reelly bout git Pickle Chin Asss BOI” is a abusive comment; however, classifiers trained using GloVe vectors were not able to classify it correctly. It is probably because words like “Fuk” and “Asss” have no embeddings. This sentence was correctly classified using *n*-gram features. The possible solution is to correct the words or to lematize them in their basic form. In addition, especially a dictionary is needed for abusive words which are not in their original form, like “Fuk” or “Fu*k”. In type II errors, comments that are labeled as non-abusive are classified as abusive by our system. For instance, “Base is missing……..all religions lead to god that says Hinduism” is correctly classified by deep neural networks with GloVe embeddings as a non-abusive comment, but it was classified as abusive with *n*-gram features. To address this issue, contextual information is required that can be achieved by using a combination of *n*-gram features.

## Discussion

There are several characteristics and limitations of the dataset that can be summarized as follows:

First, our dataset includes information such as video, video description, video title and replies of the comments. We observed that using our more precise annotation guidelines of abusive language and with additional context from our dataset, annotators were able to distinguish abusive comments easily over time. In addition, our dataset provides a complete set of information and annotations that will enable other researchers to decide how to work with it. However, in our experiments, we only used comments and replies. A few researchers, such as Obadimu et al. (2019), Mollas et al. (2020) worked on the YouTube comments only, and they did not utilize the extra information such as replies. Thus, our dataset is the first of its kind that has contextual information: replies. It also proves to be a limitation, as the comments with no replies were discarded, and only comments with replies were kept. This is also reflected in the dataset because, at the beginning of the dataset, we had 160,000 comments; however, after extracting comments that had replies, we were able to extract only 18,794 comments; see “Building the Dataset” for details.

Second, we have seen comments that had replies and these replies also had their own replies as a sub-thread. Due to the limitation of YouTube API, we were only able to extract first-level replies. As a result, we lost crucial contextual information that can be used to understand the abusive language more effectively. This is another limitation of our dataset. Finally, another relevant point to consider is that some of the comments are replies to other comments, but they have been written as a separate thread; hence, there is no automatic way to know which comments are replies to others. Detecting such information requires human evaluation of all the comments, which is way too expensive and time-consuming.

## Conclusion and future work

We have presented a dataset for abusive language detection in YouTube comments. A distinctive feature of our dataset is that the comments include replies, which provide conversational context for classification; accordingly, we called our dataset CAALDYC, standing for Context-Aware Abusive Language Detection in YouTube Comments. In particular, we gave improved annotation guidelines for abusive language detection. Our dataset, manually annotated by three experts, consists of 2,304 YouTube comments, with a total of 6,139 replies. For each comment, the dataset also includes the video, video title, and the original description.

Along with the dataset itself, we have presented a benchmark set of baseline results for further experiments on context-aware abusive language detection in YouTube comments. The baselines represent a number of classical machine-learning algorithms and deep-learning classifiers with two text representation methods. We found that, as hypothesized, the use of contextual information—the replies—improves the classification results. The best result we achieved for abusive language detection was F1 score of 91.96% with the Adaboost classifier using *n*-gram features, with the features from the context—replies—included.

In addition to being annotated with abusive language detection labels, comments in our dataset are classified into three topics: politics, religion, and other. We used this auxiliary classification to study the behavior of the classification algorithm on different topics. We also conducted experiments on single-label topic classification and achieved F1 score of 89.13% with the MLP classifier also using *n*-gram features, also with the features from the context included. Abusive language detection experiments on subsets of the dataset representing each topic class showed that the religion topic was most difficult to classify (*i.e*., the results were lower) and the other topic was the simplest, closely followed by politics.

We expect that our dataset will enable further research in identifying abusive, harmful, and hateful language in Internet and social network platforms. In addition, it can be used in a wide range of NLP applications such as public health, anxiety and depression detection, emotion detection and human reaction detection for uncertain decisions. In the future, we plan to increase the size of the dataset and include more approaches in the benchmark, especially with state-of-the-art classifiers and text representations such as BERT. Currently, we are not able to perform experiments with contextual embeddings such as BERT and ELMO due to the lack of suitable hardware. Apart from that, other researchers can use all components of the dataset including video, etc.

## Additional Information and Declarations

### Data Availability

The following information was supplied regarding data availability: The data and code for this article are available at GitHub: -Dataset: https://github.com/Noman712/contextual-abusive-language-detection/tree/main/dataset -Code: https://github.com/Noman712/contextual-abusive-language-detection/tree/main/Code.
